# Supplementary material for: Analysis of Chemically Labile Glycation Adducts in Seed Proteins: Case Study of Methylglyoxal-Derived Hydroimidazolone 1 (MG-H1)
Source: Int J Mol Sci. 2019 Jul 26;20(15):3659. doi: 10.3390/ijms20153659 (PMC6695671; doi:10.3390/ijms20153659)
Supplement: Supplementary file 1 [file ijms-20-03659-s001.pdf]

Analysis of chemically labile glycation adducts in seed proteins: a case study of methylglyoxal-derived hydroimidazolone 1 (MG-H1)

Kristina Antonova,[1,2§] Maria Vikhnina,[1,2§] Alena Soboleva,[1,2§] Tahir Mehmood,[1] Marie-Louise Heymich,[3] Tatiana Leonova,[2] Mikhail Bankin,[4] Elena Lukasheva,[2] Sabrina Gensberger-Reigl,[3] Sergei Medvedev,[4] Galina Smolikova,[4] Monika Pischetsrieder[3] and Andrej Frolov[1,2]

Supplementary information

[1]Department of Bioorganic Chemistry, Leibniz Institute of Plant Biochemistry, [2]Department of Biochemistry, St. Petersburg State University,[3]Department of Food Chemistry, Universität Erlangen-Nürnberg, and [4]Department of Plant Physiology and Biochemistry, St. Petersburg State University,

[§]These authors contributed equally in the manuscript

*Corresponding author:

Dr. Andrej Frolov

Leibniz Institute of Plant Biochemistry

Department of Bioorganic Chemistry

Weinberg 3, 06120

Halle/Saale, Germany

Tel. +49(0)34555821350

Fax. +49(0)34555821309

Directory

Protocols.. S1-4

Protocol S-1 Solid phase extraction on CHROMABOND HR-XAW weak cation exchanger cartridges.. S1-4

Protocol S-2 Solid phase extraction on CHROMABOND HR-XA strong cation exchanger cartridges.. S1-5

Protocol S-3 Solid phase extraction on CHROMABOND C18 reversed phase cartridges..S1-6

Tables.. S1-7

Table S-1 Protein recoveries and total UV densities calculated for individual pea samples separated by SDS-PAGE.. S1-7

Table S-2 Protein recoveries and total UV densities calculated for individual oilseed rape samples separated by SDS-PAGE... S1-8

Table S-3 Sensitivity and linearity parameters obtained for reference amino acids....... S1-9

Table S-4 Instrument settings applied for Orbitrap-LIT-MS and MS/MS experiments.....S1-10

Figures.. S1-11

Figure S-1 Optimization of protein yield in respect of seed material amounts, taken for phenol extraction.. S1-11

Figure S-2 SDS-PAGE electropherograms of glycated bovine serum albumin (BSA), before and after individual steps of enzymatic hydrolysis.. S1-13

Figure S-3 SDS-PAGE electropherograms of glycated bovine serum albumin, before and after individual steps of enzymatic hydrolysis... S1-15

Figure S-4 ESI-QqTOF-MS, acquired for enzymatic hydrolysate of pea protein.............S1-16

Figure S-5 Recovery of three basic amino acids in SPE procedures, performed with different CHROMABOND cartridges... S1-17

Figure S-6 Detection of individual amino acids in hydrolysates of glycated BSA prepared in presence and absence of 0.5% (w/v) sodium dodecyl sulfate (SDS)……………………..S1-18

Figure S-7 Recovery of MG-H1, arginine and phenylalanine in BSA enzymatic hydrolysates obtained in presence and absence of 0.5% (w/v) SDS with subsequent SPE…………….S1-19

Figure S-8 Recovery (%) of N^{ε}-(carboxymethyl)lysine and lysine on CHROMABOND C18 and C18 ec reversed phase cartridges..S1-21

Figure S-9 Recovery of MG-H1-d3 from the hydrolyzate of pea seed protein……......…S1-22

Figure S-10 Assessment of matrix effects, related to 20% (v/v) aqueous acetonitrile in 0.1% (v/v) formic acid (blank)………………………………………………...……………..S1-23

Figure S-11 Assessment of matrix effects, related to the pea seed protein enzymatic hydrolyzate, derivatized with L-FDVA…………………………………………….……….S1-24

Protocols

Protocol S-1 Solid phase extraction (SPE) procedure for CHROMABOND HR-XAW weak anion exchanger cartridges

Materials:

SPE cartridges: CHROMABOND HR-XA, 200 mg of material, designed for 3 ml load volume

Conditioning solution: methanol (LC-MS grade)

Equilibration solution: water

Eluent 1: 0.1 mol/L NaOH in water (MilliQ)

Eluent 2: methanol (LC-MS grade)

Eluent 3: 1% (v/v) aq. formic acid (LC-MS grade) in methanol (LC-MS grade)

Eluent 4: 5% (v/v) aq. formic acid (LC-MS grade) in methanol (LC-MS grade)

Eluent 5: 10% (v/v) aq. formic acid (LC-MS grade) in methanol (LC-MS grade)

Sample aspiration: the mixture of 146.2 μmol lysine, 1.15 μmol arginine and 1.29 μmol histidine in 1 mL of phosphate buffered saline (PBS) adjusted to pH 10.0 with 25% ammonia solution

Elution – vacuum-drived, 800-900 mbar

Procedure:

#	Step	Applied solution	Volume (mL)
1	Conditioning	Conditioning solution	5
2	Equilibration	Equilibration solution	5
3	Sample aspiration	1 mL of PBS adjusted to pH 10.0	1
4	Elution 1	Eluent 1	5
5	Elution 2	Eluent 2	2
6	Elution 3	Eluent 3	4
7	Elution 4	Eluent 4	4
8	Elution 5	Eluent 5	4

Protocol S-2 Solid phase extraction (SPE) procedure for CHROMABOND HR-XA weak anion exchanger cartridges

Materials:

SPE cartridges: CHROMABOND HR-XA, 200 mg of material, 3 ml load volume

Conditioning solution: methanol (LC-MS grade)

Equilibration solution: water

Eluent 1: methanol (LC-MS grade)

Eluent 2: water (MilliQ)

Eluent 3: 0.1 mol/L NaOH in water (MilliQ)

Eluent 4: methanol (LC-MS grade)

Eluent 5: 1% (v/v) aq. formic acid (LC-MS grade) in methanol (LC-MS grade)

Eluent 6: 5% (v/v) aq. formic acid (LC-MS grade) in methanol (LC-MS grade)

Eluent 7: 10% (v/v) aq. formic acid (LC-MS grade) in methanol (LC-MS grade)

Sample aspiration: the mixture of 146.2 µmol lysine, 1.15 µmol arginine and 1.29 µmol histidine in 1 mL of phosphate buffered saline (PBS) adjusted to pH 10.0 with 25% ammonia

Elution – vacuum-drived, 800-900 mbar

Procedure:

#	Step	Applied solution	Volume (mL)
1	Conditioning	Conditioning solution	5
2	Equilibration	Equilibration solution	5
3	Sample aspiration	1 mL of PBS adjusted to pH 10.0	1
4	Elution 1	Eluent 1	5
5	Elution 2	Eluent 2	5
6	Elution 3	Eluent 3	5
7	Elution 4	Eluent 4	2
8	Elution 5	Eluent 5	4
9	Elution 6	Eluent 6	4
10	Elution 7	Eluent 5	4

Protocol S-3 Solid phase extraction (SPE) procedure for CHROMABOND C18 reversed phase (RP) cartridges

Materials:

SPE cartridges: CHROMABOND C18 reversed phase (RP), 200 mg of material, 3 ml load

Conditioning solution: methanol (LC-MS grade)

Equilibration solution: water

Eluent 1: methanol (LC-MS grade)

Eluent 2: water (MilliQ)

Eluent 3: 0.25 mol/L ammonium acetate (LC-MS grade) in water (MilliQ)

Eluent 4: 0.1 mol/L ammonia (LC-MS grade) in water (MilliQ)

Eluent 5: 1% (v/v) ammonia (LC-MS grade), aq. 10 % (v/v) acetonitrile (LC-MS grade)

Eluent 6: 1% (v/v) ammonia (LC-MS grade), aq. 20 % (v/v) acetonitrile (LC-MS grade)

Eluent 7: 1% (v/v) ammonia (LC-MS grade), aq. 40 % (v/v) acetonitrile (LC-MS grade)

Sample aspiration: the mixture of 146.2 µmol lysine, 1.15 µmol arginine and 1.29 µmol histidine in 1 mL of phosphate buffered saline (PBS) adjusted to pH 10.0 with 25% ammonia

Elution – vacuum-drived, 800-900 mbar

Procedure:

#	Step	Applied solution	Volume (mL)
1	Conditioning	Conditioning solution	5
2	Equilibration	Equilibration solution	5
3	Sample aspiration	1 mL of PBS adjusted to pH 10.0	1
4	Elution 1	Eluent 1	5
5	Elution 2	Eluent 2	5
6	Elution 3	Eluent 3	5
7	Elution 4	Eluent 4	5
8	Elution 5	Eluent 5	4
9	Elution 6	Eluent 6	4
10	Elution 7	Eluent 5	4

Tables

Table S-1 Protein recoveries and total UV densities calculated for individual pea samples separated by SDS-PAGE

Sample	Sample weight (mg)	Protein concentration (mg/mL)	Protein recovery (mg/g fresh weight)	UV densities (AU)[a]
Pea-1	49.5	38.1	96.2	38200
Pea-2	50.4	39.3	97.5	36200
Pea-3	51.6	42.4	102.8	34100
Pea-AA-5d-1	51.4	32.8	87.8	34100
Pea-AA-5d-2	50.3	54.9	129.8	35500
Pea-AA-5d-3	50.3	35.1	91.6	38000

Pea and Pea-AA-5d denote the seeds of yellow-seeded cultivar Millennium before and after accelerated ageing (AA) during five days, respectively; AU, arbitrary units

Table S-2 Protein recoveries and total UV densities calculated for individual oilseed rape samples separated by SDS-PAGE

Sample	Sample weight (mg)	Protein concentration (mg/mL)	Protein recovery (mg/g fresh weight)	UV densities (AU)[a]
Brassica-1	209.4	73,93	103,50	29315
Brassica-2	204.7	77,34	108,28	28814
Brassica-3	213.1	70,42	98,59	28361
Brassica-4	209.7	68,64	96,10	28075
Brassica-5	210.2	85,31	119,44	28361
Brassica-NA-1	211.5	58,64	82,10	28326
Brassica-NA-2	212.9	38,50	53,90	28837
Brassica-NA-3	203.6	58,67	82,14	29695
Brassica-NA-4	207.3	67,46	94,44	30906
Brassica-NA-5	207.2	80,31	112,44	30126

Brassica and Brassica-NA denote the seeds of the oilseed rape cultivar Oredezh-2 (K-4917) after one and nine years of natural ageing (dark, 18° C), respectively; AU, arbitrary units

Table S-3 Sensitivity and linearity parameters obtained for reference amino acids

Analyte	*m/z*	t_R	LOD (fmol)	LOQ (pmol)	LDR	Slope	Intercept	R^2
Arginine[a]	455.20	11.8	5.0	0.1	1.0E+03	1.0E+06	2.0E+06	0.999
Lysine[a]	427.19	11.6	10.0	0.1	1.0E+03	1.2E+04	-1.0E+04	0.988
Alanine[a]	370.14	13.7	10.0	0.025	2.0E+03	1.4E+04	1.1E+04	0.988
Phenylanine[a]	446.17	17.6	25.0	0.5	0.1E+03	3.0E+04	8.1E+04	0.993

[a]serial dilutions were prepared in 20% acetonitrile

Table S-4 Instrument settings applied for Orbitrap-LIT-MS and MS/MS experiments

Parameter	Setting
MS conditions	
Ionization mode	Positive
Mass analyzer	LIT-Orbitrap (FT-scan)
Ion spray voltage (IS)	4.0 kV
Nebulizer gas	35 psig
Auxillary gas	30 psig
Capillary temperature	275 °C
Mass to charge ratio (*m/z*) range	400 – 2000
Resolution	30000
MS/MS conditions	
Ionization mode	Positive
Mass analyzer	LIT-Orbitrap (FT-scan)
Ion spray voltage (IS)	4.0 kV
Fragmentation	Collision activated dissociation
Isolation width	2 Da
Charge state rejected	1+
Normalized collision energy	35%
Activation frequency	0.25
Activation time	10 ms

Figures

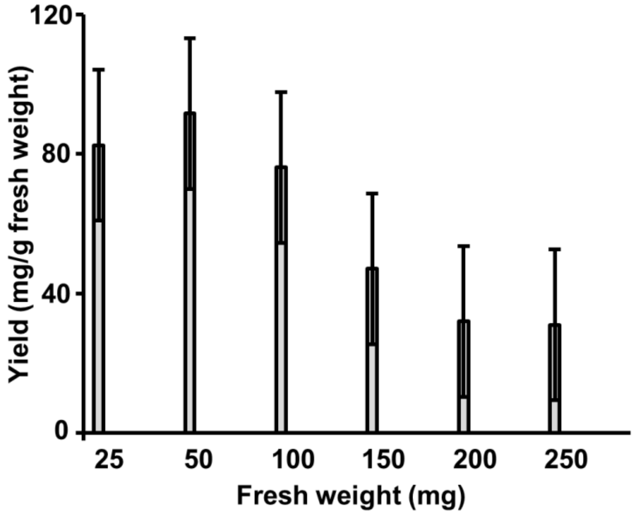

Figure S-1 Optimization of protein yield in respect of seed material amounts, taken for phenol extraction

A

St BH1 AH1 BHS1 AHS-1 St BH2 AH2 BHS2 AHS2

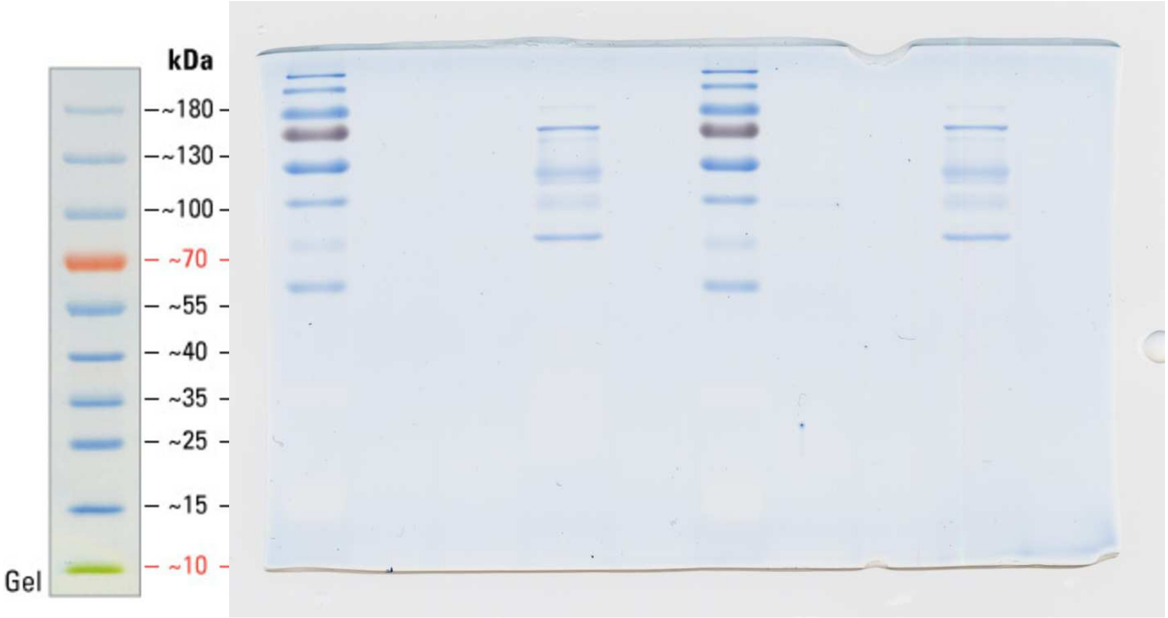

B

St BH1 APE1 APES1 APK-1 AH1

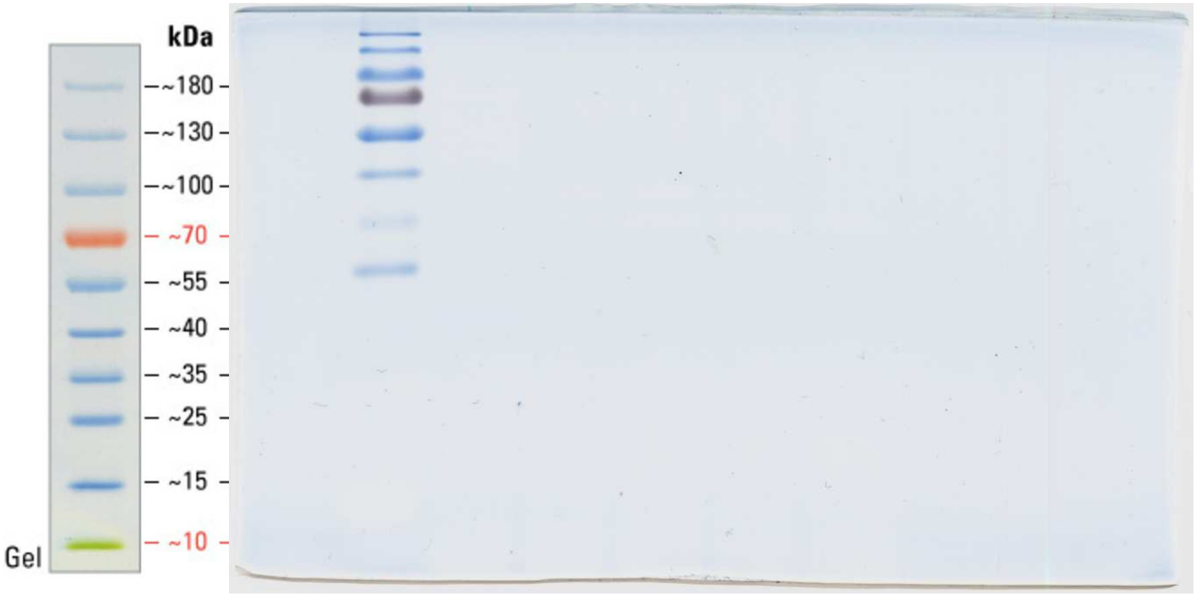

C

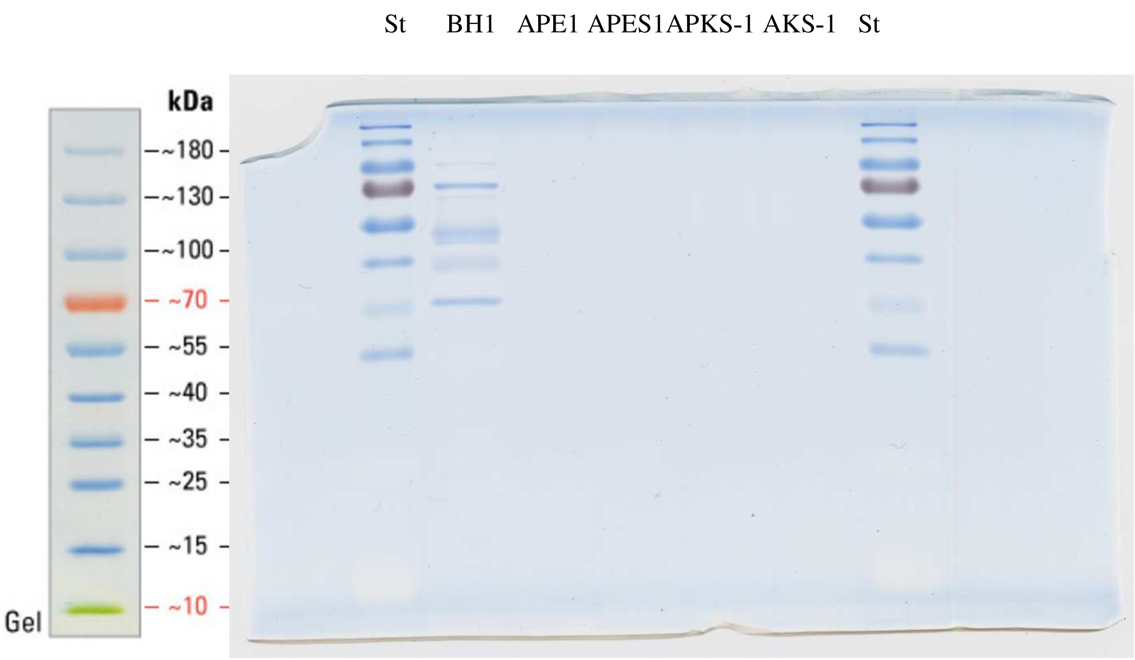

Figure S-2 SDS-PAGE electropherograms of glycated bovine serum albumin (BSA), before and after individual steps of enzymatic hydrolysis. Hydrolysis was performed in triplicates. The aliquots (5 µg) of all samples were loaded on a gel in 10 µL of sample buffer. BH, before hydrolysis; APE, after incubation with Pronase E; APK, after incubation with Proteinase K; AH, after complete hydrolysis; BHS, before hydrolysis in presence of 0.5% (w/v) SDS; APES, after incubation with Pronase E in presence of 0.5% (w/v) SDS; APK, after incubation with Proteinase K in presence of 0.5% (w/v) SDS; AH, after complete hydrolysis in presence of 0.5% (w/v) SDS; St, Page Ruler Prestained Protein Ladder

A

St BH1 AH1 BHS1 AHS-1 St BH2 AH2 BHS2 AHS2

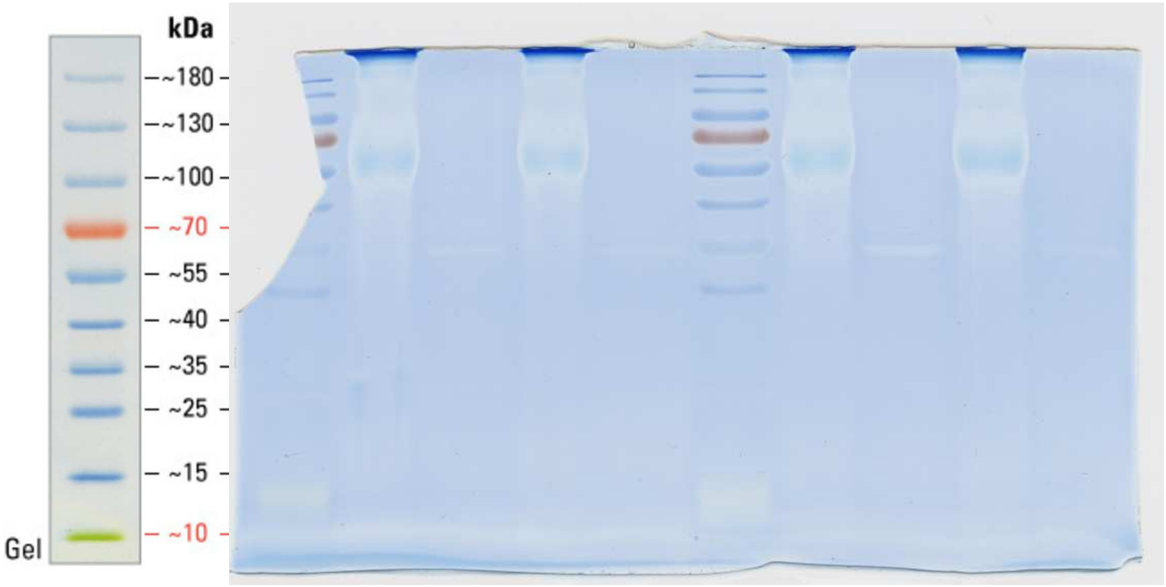

B

St BH1 APE1 APE1 APK-1 AH-1 BH-3AH3BH4AH4

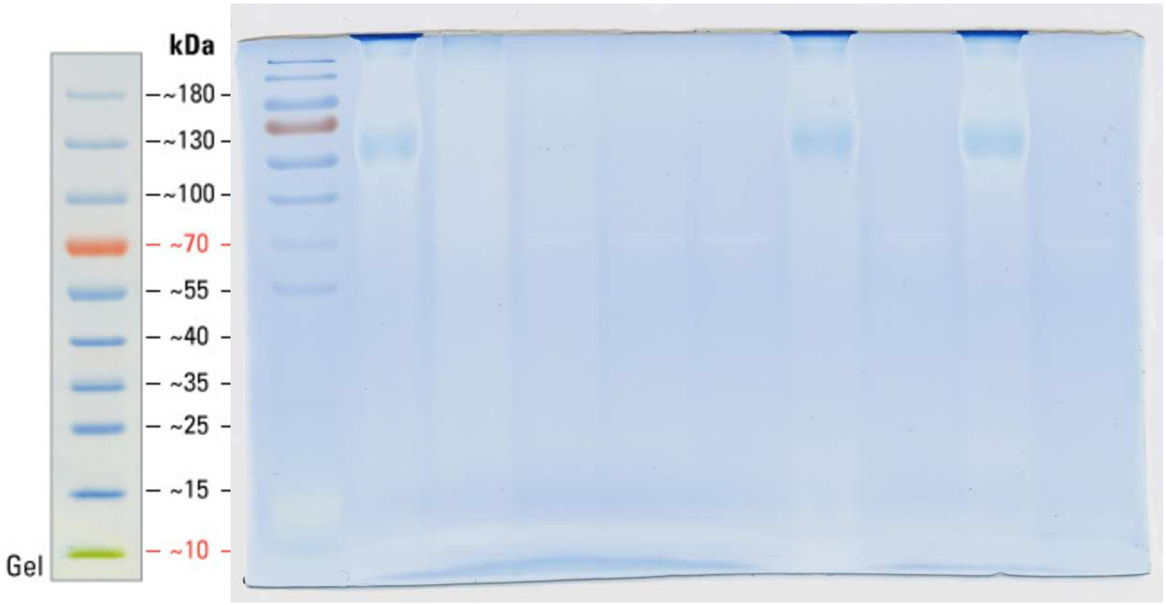

C

St BHS1 APES1 APES1 APKS-1AHS-1

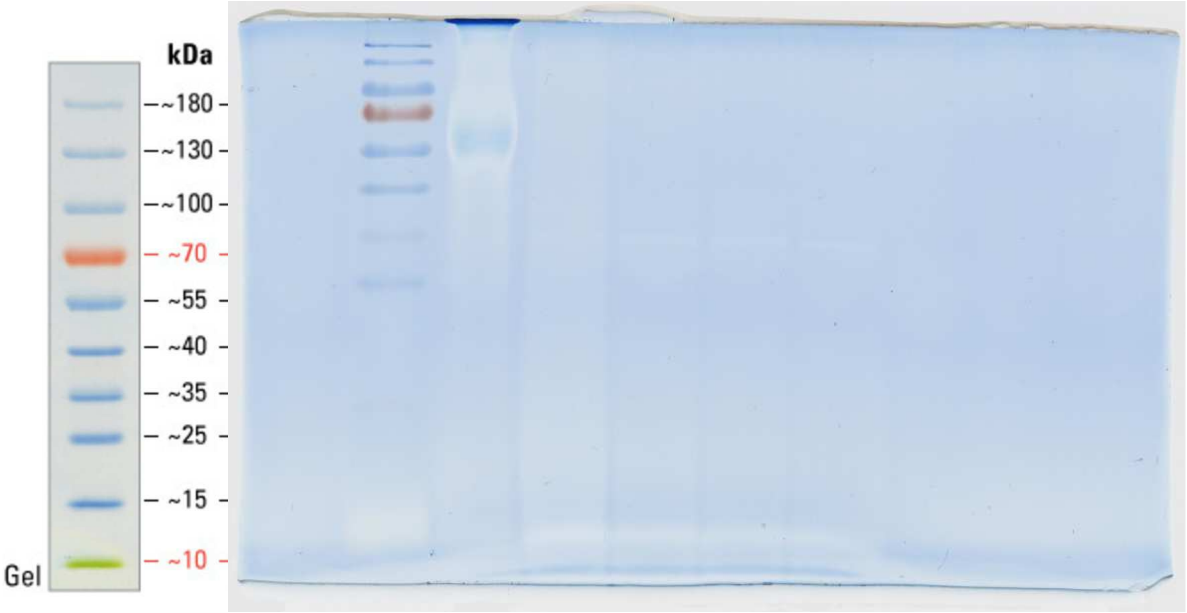

Figure S-3 SDS-PAGE electropherograms of glycated bovine serum albumin (HSA), before and after individual steps of enzymatic hydrolysis. Hydrolysis was performed in triplicates. The aliquots (5 µg) of all samples were loaded on a gel in 10 µL of sample buffer. BH, before hydrolysis; APE, after incubation with Pronase E; APK, after incubation with Proteinase K; AH, after complete hydrolysis; BHS, before hydrolysis in presence of 0.5% (w/v) SDS; APES, after incubation with Pronase E in presence of 0.5% (w/v) SDS; APK, after incubation with Proteinase K in presence of 0.5% (w/v) SDS; AH, after complete hydrolysis in presence of 0.5% (w/v) SDS; St, Page Ruler Prestained Protein Ladder

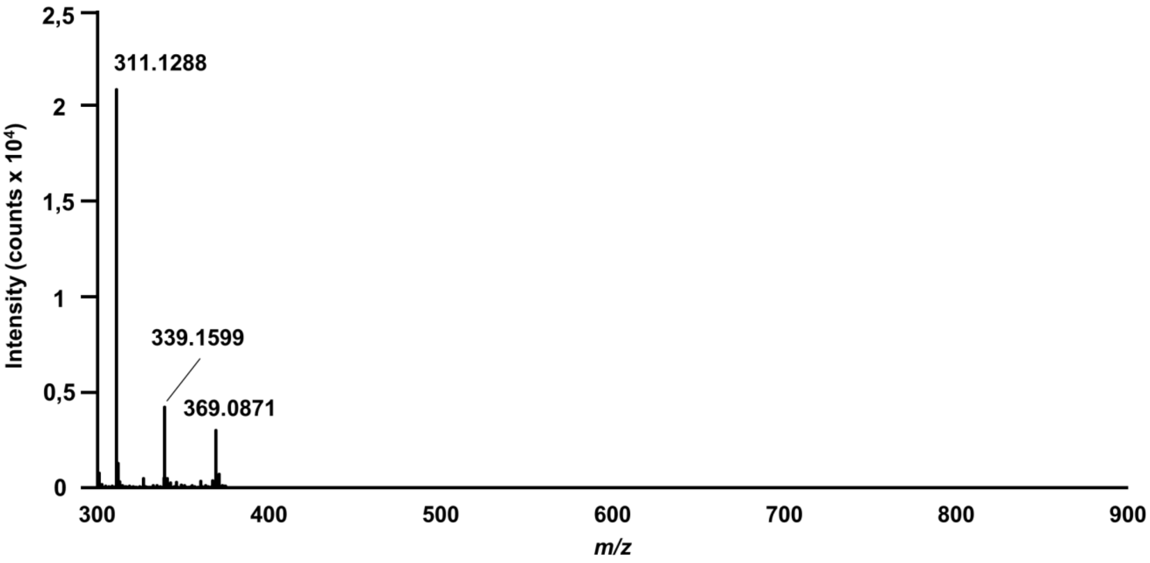

Figure S-4 ESI-QqTOF mass spectrum, obtained for enzymatic hydrolysate of pea protein. The spectrum was acquired by a syringe infusion (10 µL/min) in a QqTOF-MS (Triple TOF, Sciex, Darmstadt, Germany), operated in positive ion mode. No signals of multiply charged ions could be observed in the spectrum that indicated completeness of hydrolysis.

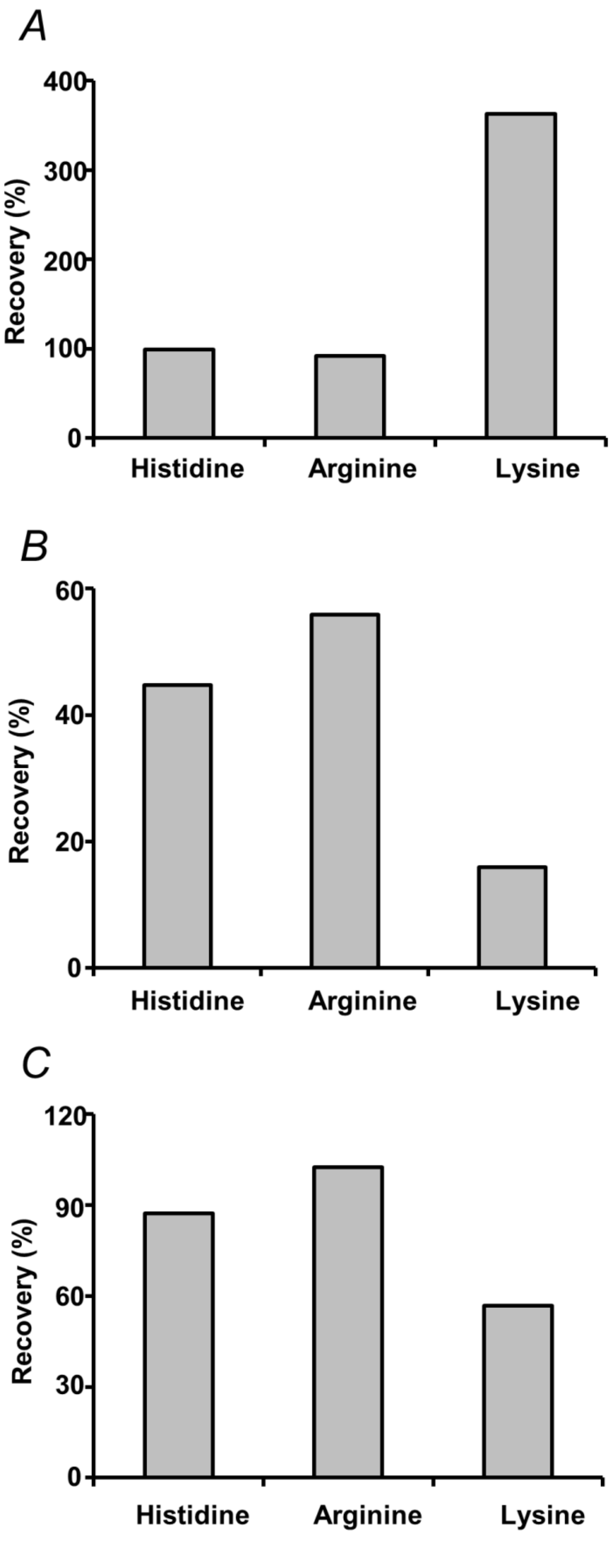

Figure S-5 Recovery of three basic amino acids from weak anion exchanger CHROMABOND HR-XAW (A), strong anion exchanger CHROMABOND HR-XA (B) and reversed phase CHROMABOND C18 (C) cartridges (according the Protocols S1-1, 2 and 3)

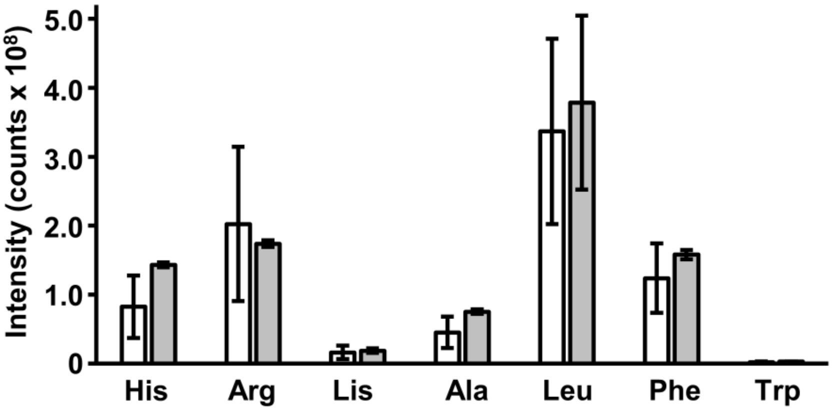

Figure S-6 Detection of individual amino acids in hydrolysates of glycated BSA (1 mg/mL) prepared in presence (white) and absence (grey) of 0.5% (w/v) sodium dodecyl sulfate (SDS). After completion of hydrolysis, SDS was removed by SPE on CHROMABOND C18 ec cartridges, as described in the material and method part. Then, 20 µg of the pre-cleaned hydrolyzates were derivatized with L-FDVA and 2.7 µg were analyzed by UHPLC-ESI-LIT-Orbitrap-MS. Relative abundances of analytes were obtained by integration of corresponding LC-MS extracted ion chromatograms (XICs) at *m/z* 436.16, 455.20, 427.19, 370.14, 412.18, 446.17 and 485.18 for the L-FDVA derivatives of histidine, arginine, lysine, alanine, leucine, phenylalanine and tryptophan, respectively.

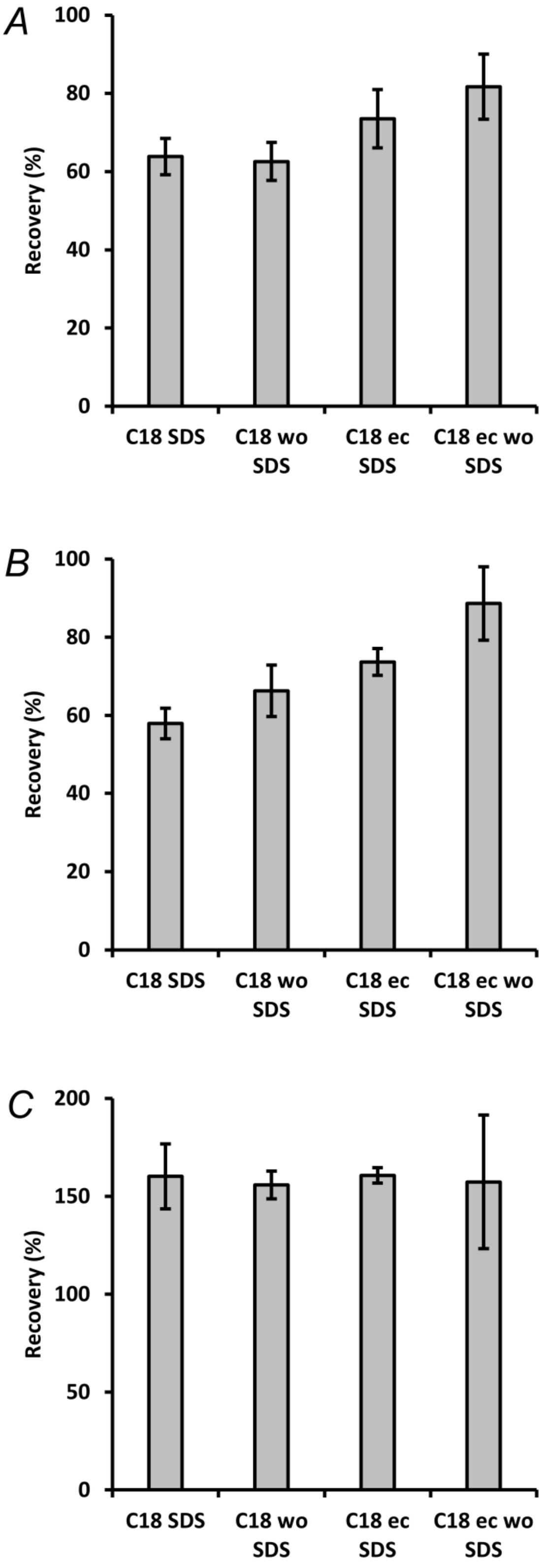

Figure S-7 Recovery (%) of MG-H1 (A), arginine (B) and phenylalanine (C) in enzymatic hydrolysates obtained with 1 mg/mL glycated BSA in presence and absence of 0.5% (w/v) SDS with subsequent SPE on CHROMABOND C18 or C18

ec cartridges. Relative abundances of analytes were obtained by integration of corresponding LC-MS extracted ion chromatograms (XICs) at *m/z* 509.21, 455.20 and 446.17 ± 0.03 for the L-FDVA derivatives of MG-H1, arginine, and phenylalanine, respectively. The obtained values were related to abundances observed without application of SDS and SPE and expressed in %.

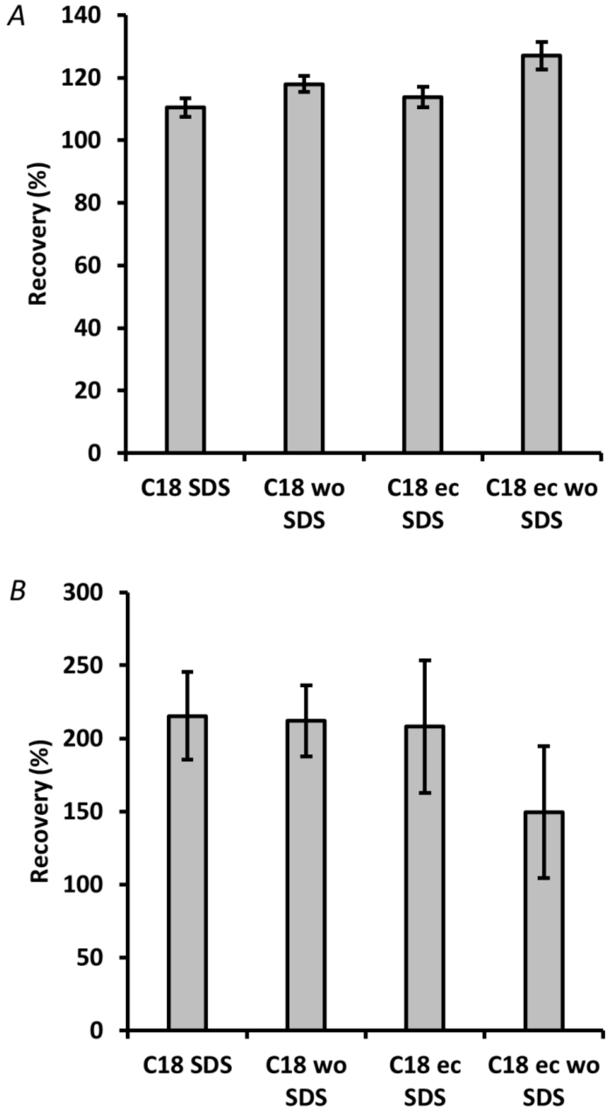

Figure S-8 Recovery (%) of N^{ε}-(carboxymethyl)lysine (CML, A) and lysine (B) from enzymatic hydrolysates obtained with 1 mg/mL glycated BSA in presence and absence of 0.5% (w/v) SDS with subsequent SPE on CHROMABOND C18 and C18 ec cartridges. Relative abundances of analytes were obtained by integration of corresponding LC-MS extracted ion chromatograms (XICs) at *m/z* 485.20 and 427.19 for the L-FDVA derivatives of CML and lysine, respectively, and related to abundances observed without application of SDS and SPE.

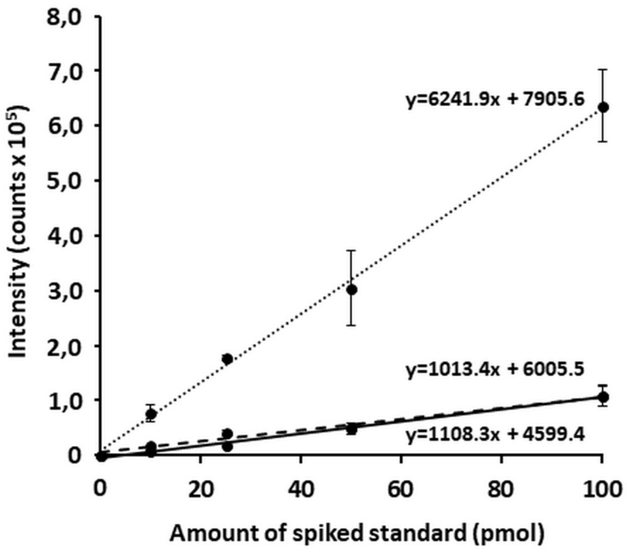

Figure S-9 Recovery of MG-H1-d3 standard from the hydrolyzate of pea seed protein (solid line) in comparison to the same procedure performed without addition of the pea seed protein (dashed line) and to the standard solution of MG-H1-d3 dissolved in phosphate buffered saline (PBS) and directly subjected to LC-MS (dotted line). For recovery experiments, 3.75 – 37.5 µL of 50 µmol/L MG-H-d3 solution in PBS was supplemented to 300 µg aliquots of protein in 1 mL PBS solution containing 0.16% (v/v) aq. SDS. After completion of hydrolysis and removal SDS by solid phase extraction (according to the protocols described in Material and method part), the SPE eluates were dried, reconstituted in 50 µL of 20% (v/v) aq. acetonitrile, and 20 µL of samples containing spiked MG-H1-d3 were derivatized with N^2-(5-fluoro-2,4-dinitrophenyl)-*L*-valine amide (L-FDVA) and analyzed by RP-UHPLC-LIT-Orbitrap-MS in parallel with the same amounts of standard MG-H1-d3 in PBS.

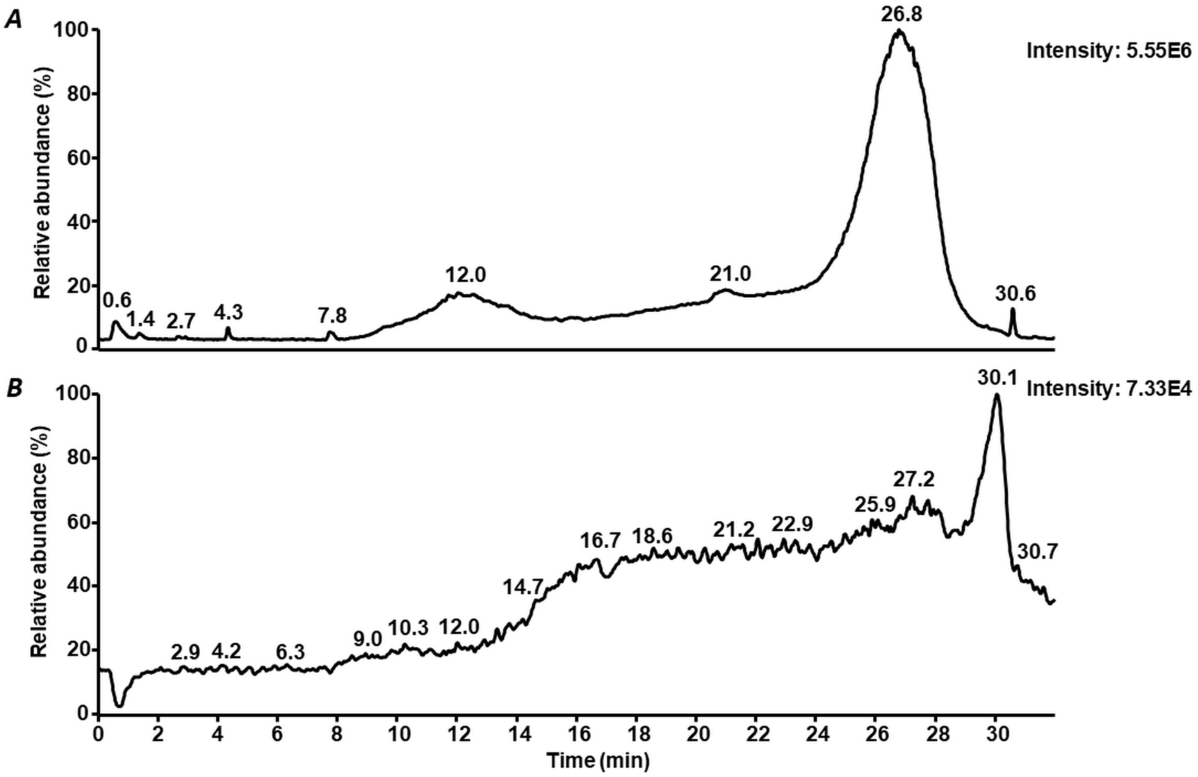

Figure S-10 Assessment of matrix effects, occurring during liquid chromatography-electrospray ionization mass spectrometry (LC-ESI-MS) experiments, performed with 25 µL of 20% (v/v) aqueous acetonitrile in 0.1% (v/v) aq. formic acid (blank), injected in a Dionex Ultimate 3000 UHPLC System, equipped with a reversed phase Hypersil GOLD aQ column (100 x 1 mm, 1.9 µm particle size), coupled on-line to a hybrid LIT-Orbitrap mass spectrometer, operated in a positive ion high resolution mode and controlled by Xcalibur software (all from Thermo Fisher Scientific, Bremen, Germany). MG-H1-d3 (0.2 pmol/µL) was continuously infused (3 µL/min) with a syringe pump model 22 (Harvard apparatus, Germany) in the column effluent flow via a T-connection installed post-column prior to a high temperature electrospray ionization (HESI) ion source. Total ion current (TIC, A) and characteristic extracted ion chromatogram (XIC) for MG-H1-d3 at *m/z* = 512.23 ± 0.01 (B) were recordered.

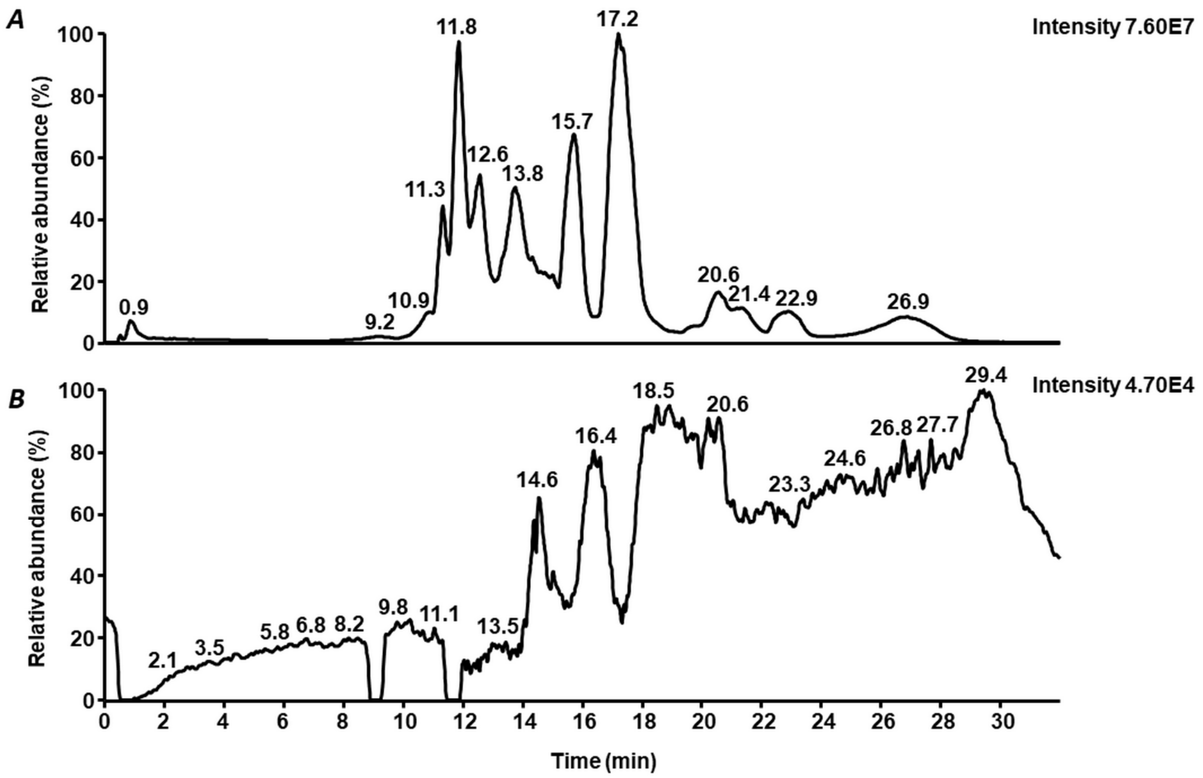

Figure S-11 Assessment of matrix effects, occurring during liquid chromatography-electrospray ionization mass spectrometry (LC-ESI-MS) experiments, performed with pea seed protein hydrolyzate (1 mg/mL), obtained and purified by solid phase extraction (SPE) as described in Material and method section. Dried SPE eluates were reconstituted in 20% (v/v) aqueous acetonitrile, derivatized with N^2-(5-fluoro-2,4-dinitrophenyl)-*L*-valine amide (L-FDVA), and 25 µL of derivatized hydrolyzate samples (corresponding to 13.6 µg of protein) were injected in a Dionex Ultimate 3000 UHPLC System, equipped with a reversed phase Hypersil GOLD aQ column (100 x 1 mm, 1.9 µm particle size), coupled on-line to a hybrid LIT-Orbitrap mass spectrometer, operated in a positive ion high resolution mode and controlled by Xcalibur software (all from Thermo Fisher Scientific, Bremen, Germany). MG-H1-d3 (0.2 pmol/µL) was continuously infused (3 µL/min) with a syringe pump model 22 (Harvard apparatus, Germany) in the column effluent flow via a T-connection installed post-column prior to a high temperature electrospray ionization (HESI) ion source. Total ion current (TIC, A) and characteristic extracted ion chromatogram (XIC) for MG-H1-d3 at *m/z* = 512.23 ± 0.01 (B) were recordered.
